# Case report: Laparoscopic-assisted distal gastrectomy for gastric cancer in a patient with situs inversus totalis

**DOI:** 10.3389/fsurg.2023.1090910

**Published:** 2023-03-21

**Authors:** Kaifeng Zhu, Qiang Hu, Yuanshui Sun

**Affiliations:** ^1^ The Second School of Clinical Medicine, Zhejiang Chinese Medical University, Hangzhou, China; ^2^ Department of General Surgery, Tongde Hospital of Zhejiang Province, Hangzhou, China

**Keywords:** gastric cancer, gastrectomy, situs inversus totalis (SIT), case report, surgery

## Abstract

**Background:**

Situs inversus totalis (SIT) is a rare congenital disease with a series of clinical features characterized by a mirror image distribution of the viscera to the normal anatomy.

**Case presentation:**

This study aims to report a 63-year-old male SIT patient with gastric cancer with a preoperative diagnosis of stage IIB gastric cancer (cT3N0M0), who underwent a preoperative multi-disciplinary treatment (MDT) discussion and an abdominal enhancement CT for visceral evaluation to ensure a successful operation. A laparoscopic-assisted distal gastrectomy including D2 lymph node dissection and Billroth I reconstruction was successfully performed. Laparoscopic radical gastric gastrectomy and D2 lymph node dissection were performed through the opposite surgical station to the conventional one, followed by digestive tract reconstruction under small incision-assisted direct vision. There was less blood loss throughout the operation, no postoperative complications, and the patient was discharged successfully 10 days after surgery. Histopathological examination showed ulcerated high-medium differentiated adenocarcinoma stage IB (PT2N0M0). There were no complications or tumor recurrence in the patient with examination 6 months after the operation.

**Conclusion:**

Surgery in a patient with gastric cancer and SIT can be safely performed by the application of 3D laparoscopy and small incisions to assist the digestive tract reconstruction under direct vision.

## Introduction

Situs inversus totalis (SIT) is a rare autosomal recessive disorder occurring in 1 in 5,000–20,000 people and is characterized by a mirror image distribution of the viscera to the normal anatomy (1). However, due to the small number of cases, most surgeons have no surgical experience in this type of disease, which makes the operation difficult. Most of the treatment for this type of operation is mainly reflected in the removal of the gallbladder and appendix (2, 3). Looking back over the past few decades, there are very few reports on the surgical treatment of patients with total inverse gastric cancer.

As early as 1936, Yamaguchi et al. (4) reported the first case of SIT with gastric cancer in the world, and the first laparoscopic surgery in SIT patients with gastric cancer was not reported until 2003 when Yamaguchi et al. (5) reported the first laparoscopic-assisted distal gastrectomy. At present, there are still few case reports on laparoscopic radical gastrectomy for patients with gastric cancer in total inversion.

This study reports the case of an SIT patient with gastric cancer in the sinus region who underwent a successful radical gastrectomy and recovered well with no recurrence and no serious complications in the examination 6 months after the operation. This study was reported in agreement with principles of the CARE guidelines (6).

## Case presentation

**Figure 1:**
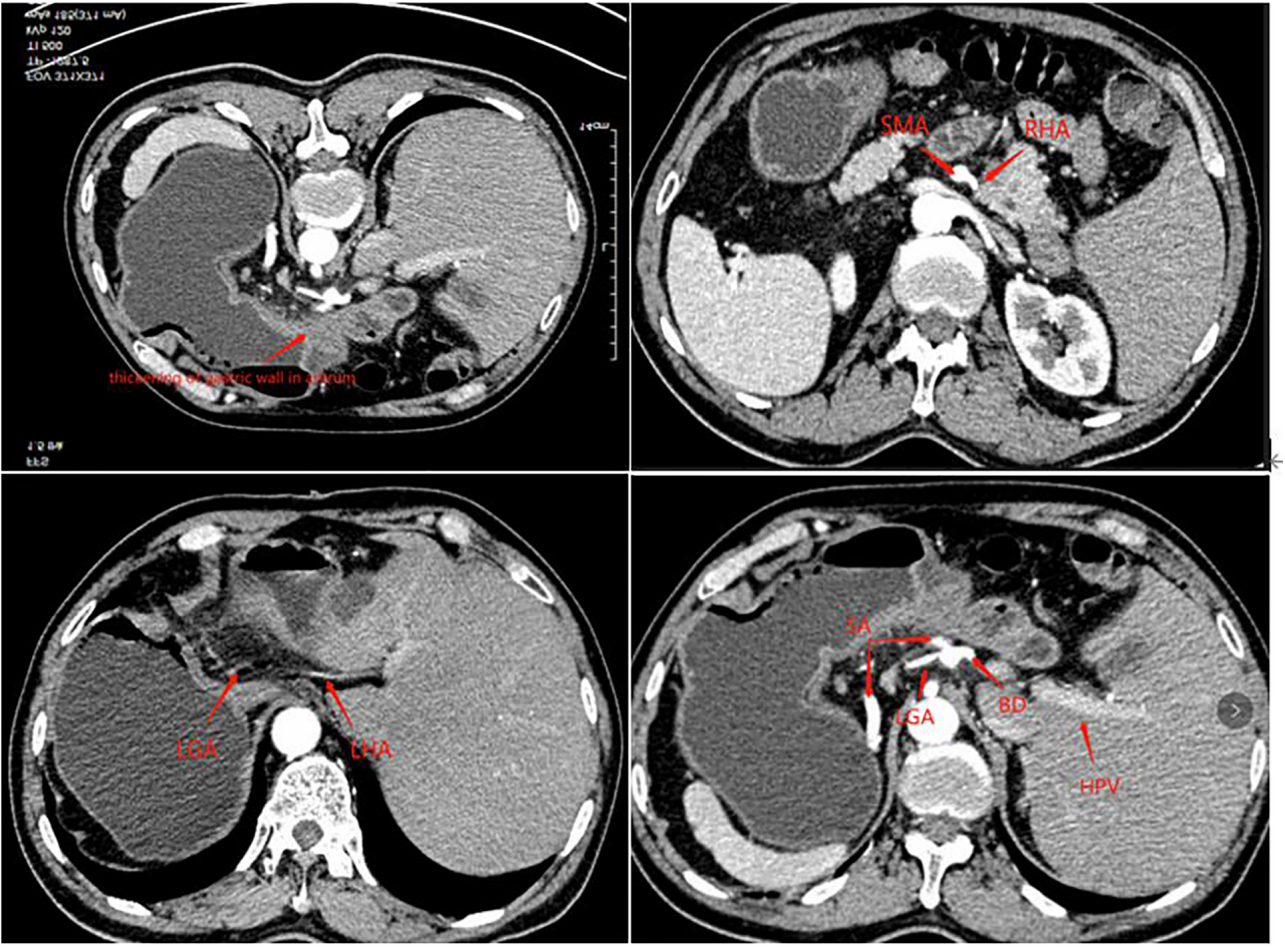
Enhanced CT arterial phase shows vascular variation.

A 63-year-old man presented to our outpatient clinic complaining of epigastric pain for 2 months. Gastroscopy showed an ulcerative lesion in the gastric sinus, located on the lesser curvature of the gastric antrum, about 2.5 cm × 1.2 cm in size. Biopsy pathology showed high-medium differentiated adenocarcinoma, and the perfected CT of the patient showed horizontal turning of the intra-abdominal organs and local thickening of the gastric wall in the sinus with enlargement of small adjacent lymph nodes. Enhanced CT examination showed hepatic vascular variation. The common hepatic artery extended directly to bridge the gastroduodenal artery (GDA) without the proper hepatic artery, the right hepatic artery (RHA) emanated from the superior mesenteric artery (SMA), and the left hepatic lobe is supplied by the accessory left hepatic artery (ALHA) from the left gastric artery (LGA), and the celiac trunk and portal system were normal (Figure 1). The preoperative examination did not reveal any operational contraindications for the patient. After inviting the medical oncology department and radiotherapy department for the multi-disciplinary treatment (MDT) discussion, the patient was diagnosed with gastric cancer CT3N0M0 (stage IIB). Considering that the tumor was small and no obvious lymph node metastasis was suggested by imaging, the patient could be operated on with radical resection of the tumor although he had local vascular variation, which did not affect the surgical operation. Finally, D2 surgical resection was directly performed on the patient without preoperative adjuvant chemotherapy in a combination of the patient's age and economy, followed by laparoscopic-assisted distal gastrectomy and D2 lymph node dissection and Billroth I anastomosis in this patient.

**Figure 2:**
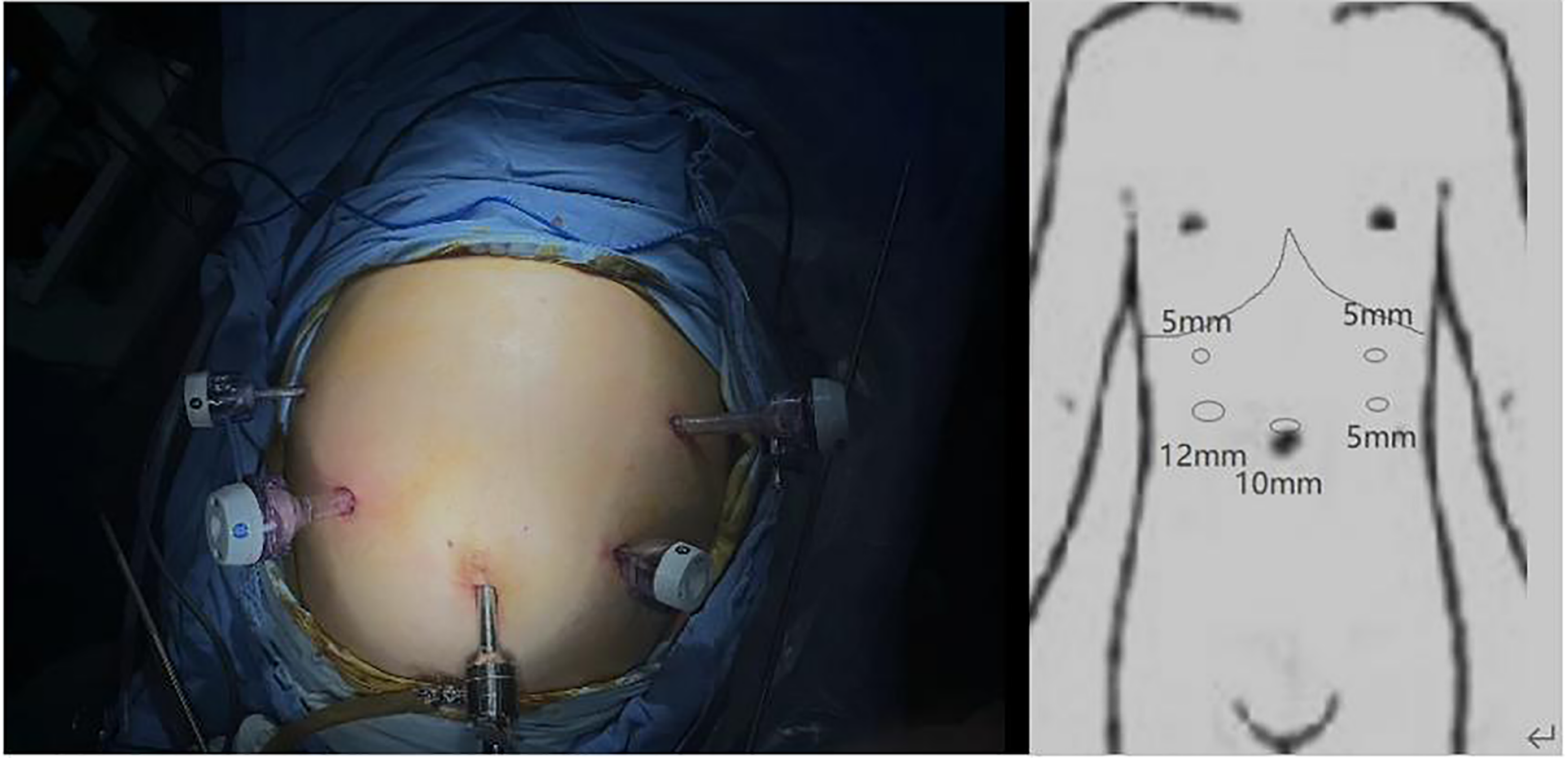
Perforation position.

For the surgical approach, we adopted the opposite stance and perforation to conventional gastric cancer surgery (Figure 2) and used a 3D laparoscope to allow a more detailed view of the anatomical levels. The patient was placed in a supine split-legged position, and the lead surgeon operated on the right side of the patient. A 10 mm trocar was placed in the umbilicus and a camera was put in to explore that there were no malignant ascites in the abdominal cavity and the tumor did not break through the surface of the gastric sinus, but in the right midclavicular line in the flat umbilical position. A 12 mm trocar was placed and three 5 mm trocars were placed on the remaining side. The gastric colon ligament was separated by an ultrasonic blade at the upper edge of the transverse colon, followed by free cutting of the splenic colic ligament and splenic ligament to the splenic hilum to the right, dissection of the left vascular of the gastric omentum and the short gastric vessels, clearing of station 4 lymph nodes, and stripping of the anterior lobe of the transverse mesocolon. The posterior wall of the gastric antrum was excised from the transverse mesocolon, which then was dissected upward to the superior margin of the pancreas and separated to the right to the lower margin of the duodenal bulb, followed by separation and ligation of the right gastroepiploic vein and the right gastroepiploic artery, and clearing of station 6 lymph nodes. After that, the gastric body and greater omentum were turned cephalad, and the accessory left hepatic artery was ligated and dissected after separating the left gastric artery and protecting, followed by the clearing of the lymph nodes of station 7 and station 9, the clearing of station 8 lymph nodes along the common hepatic artery to the left, and the clearing of station 11 lymph nodes along the splenic artery to the right. The stomach and greater omentum were then turned caudally, and the hepatoduodenal ligament was opened over the pylorus to find the right gastric artery, which was dissected at the root of its confluence with the common hepatic artery, and station 5 lymph nodes were cleared, freeing the superior duodenum by about 3 cm; the hepatogastric ligament was dissected immediately over the liver to the right side of the cardia and the lymph nodes of station 1 and station 3 were cleared, and the 12 groups of lymph nodes were cleared over the common hepatic artery.

**Figure 3:**
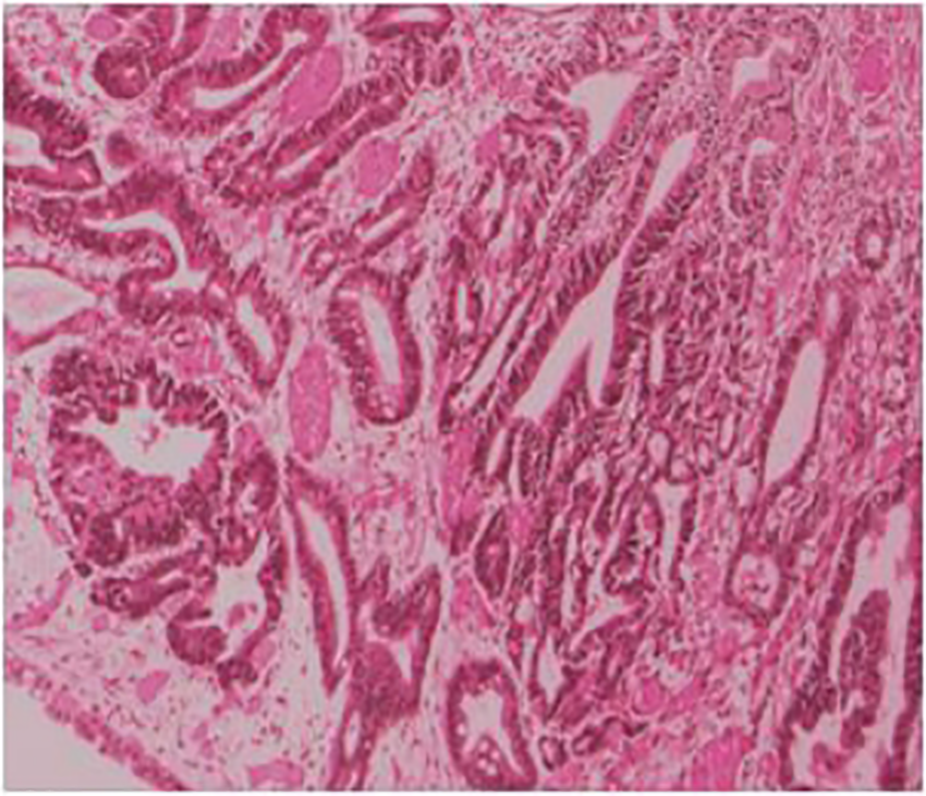
Ulcerated high-medium differentiated adenocarcinoma (2 cm × 3 cm), muscular infiltration.

The postoperative pathology showed ulcerated high-medium differentiated adenocarcinoma (2 cm × 3 cm) and muscular infiltration, without nerve involvement, and the examination results of upper and lower margins were all negative. No cancerous tissue was seen in the omental tissue, 14 lymph nodes in the small curved side did not show cancer metastasis, and 6 lymph nodes in the large curved side did not show cancer metastasis (Figure 3). The postoperative diagnosis was gastric cancer (PT2N0M0 stage IB).

The patient recovered well after surgery and was discharged 10 days after surgery, with no serious complications, abnormal liver function, and obvious tumor recurrence after 6 months of follow-up.

## Discussion

SIT is an autosomal recessive disease, but its exact genetic cause remains unknown. The inhibition of extraembryonic fluid flow during embryogenesis is associated with cardiovascular abnormalities; familial long QT syndrome; hand, foot and mouth disease; and various urinary abnormalities (7, 8). In the available case reports (8–11), four cases were clearly reported in which patients with gastric cancer presented with varying degrees of vascular or organ degeneration. Therefore, we also performed relevant tests in the preoperative examination and evaluation of SIT patients, and we also performed an enhanced CT abdomen before surgery. Fortunately, there was no significant cardiovascular disease in the patient. Although there were variants in both the left and right hepatic arteries in the patient, there were no significant variants in the course of the named vessels around the stomach that needed to be dissected during gastric cancer surgery as well as the anatomy of the surrounding organs; we could safely perform surgery on the patient with gastric cancer.

There is no difference in the treatment for SIT patients with gastric cancer from patients with normal gastric cancer, but there are some difficulties in the surgical treatment. First, the organs are distributed in a mirror image flip from the normal anatomical structures and there may be local vascular variants. When the camera of the laparoscope is drawn closer to magnify the surgical field, it can easily lead to confusion about anatomical structures due to the limitation of the field of view. Second, it requires a lead surgeon who is skilled in gastric cancer surgery as the lead surgeon needs to change his operating habits from the customary right-handed operation to a left-handed operation, and it may be confusing due to the reversal of the patient's anatomy (12), thus increasing the difficulty of the operation. Therefore, we chose 3D laparoscopy to present the patient's intra-abdominal organs in three dimensions so that the lead surgeon could distinguish the anatomical structures more intuitively. Third, the surgical assistant will operate in the opposite way to the normal gastric operation due to the adjustment of the standing position, and there may be poor cooperation with the lead surgeon.

As there is no clear standard for surgical staff positioning in the available literature, we chose the opposite standing position from the conventional surgery for this laparoscopic surgery, in which the operator was on the right side of the patient. We believe that it is effective to change the surgeon's standing position during the surgery of SIT patients, and such a change can avoid the confusion of anatomical structures due to the mirror image change of the organ's position to some extent. At the same time, the incision was mirrored for the convenience of the surgeon, and the 12 mm trocar was switched from the normal left subcostal margin to the flat umbilical point on the right midclavicular line.

The operative steps were performed according to the standardized D2 lymph node dissection technique for distal gastric cancer, with a gradual intraoperative transition according to the dissection area. At the level of lymph node dissection, there was no significant difference between the mirror organ and normal anatomy, and it should be noted that the spleen was located on the left side of the patient due to the inversion of the organ; we should, therefore, avoid damaging the spleen and splenic artery when performing 4.11 lymph node dissection. Although we have the sufficient technical ability to perform total laparoscopic distal gastrectomy, we finally chose to perform Billroth I reconstruction under direct vision through an epigastric incision to ensure the safety of the anastomosis due to the discomfort brought by the backhand operation. The reconstruction under direct vision can change the laparoscopic backhand operation for digestive tract reconstruction into a familiar routine reconstruction by changing the position of the lead surgeon to ensure the safety of the anastomosis and prevent postoperative leakage or obstruction, and the patient was discharged on the 10th postoperative day with no significant postoperative complications.

## Conclusion

SIT patients with gastric cancer are very rare and may have vascular variants, so it is important to refine the preoperative evaluation. Also, the application of 3D laparoscopy can reduce the confusion caused by the opposite position of organs by showing the anatomical structures in three dimensions due to the mirror image flip of the organs, while small incisions to assist the digestive tract reconstruction under direct vision can also avoid the backhanded reconstruction of the digestive tract under laparoscopy, thus increasing the operation safety.

## Data Availability

The original contributions presented in the study are included in the article/**Supplementary Material**, further inquiries can be directed to the corresponding author.
